# US e-learning course adaptation to the Ukrainian context: lessons learned and way forward

**DOI:** 10.1186/s12909-018-1349-1

**Published:** 2018-11-01

**Authors:** Ann Downer, Anna Shapoval, Olga Vysotska, Iryna Yuryeva, Tetiana Bairachna

**Affiliations:** 1 International Training and Education Center for Health (I-TECH), Department of Global Health, University of Washington, 908 Jefferson Street, Seattle, WA 98104 USA; 2 International Training and Education Center for Health (I-TECH) Ukraine, Kyiv, Ukraine; 3 Ukrainian Family Medicine Training Center, Bogomolets National Medical University, Kyiv, Ukraine

**Keywords:** Continuing medical education, Distance learning, E-learning, Leadership and management, Transition to local ownership, Sustainability of development investments

## Abstract

**Background:**

Access to continuing education opportunities is limited for Ukrainian healthcare workers, and the need is acute in order to support healthcare reform efforts currently underway in Ukraine. Online learning is a cost-effective mechanism for continuing education since healthcare workers can remain on the job during training. It also provides a means of keeping health professionals up to date on their knowledge and skills in rapidly changing and increasingly complex healthcare environments.

**Methods:**

This paper describes the process of adapting an existing e-learning course from a US institution to the Ukrainian setting. Course participants’ feedback was used to evaluate the effectiveness of the adapted version that was piloted twice in 2016–2017 with 53 participants in total, 46 of whom completed the course and contributed to the evaluation.

**Results:**

This was the first fully online course on Leadership and Management in Health (LMiH) to be offered in Ukraine. Several lessons were learned during course adaptation when multiple aspects of the Ukrainian environment were taken into account including 1) linguistic accessibility, 2) access to the Internet, 3) computer literacy, and 4) novelty of online learning. Based on these findings, course material was first adapted by translating it from English to Ukrainian with the emphasis on cultural adjustment of idioms and real life examples. Then, using the first pilot results and participants suggestions, videotaped interviews with local healthcare management experts were added in order to further enhance cultural suitability as well as relevance and applicability of the course concepts. The last but not least lesson learned consisted in the fact that enhancing, transitioning, and sustaining online learning to new contexts required engagement of key stakeholders, national level support, and technical assistance through implementation and beyond yet turned out to be both cost-effective and sustainable investment of limited resources. Formative evaluation confirmed that the adaptation efforts resulted in a course relevant and acceptable to healthcare professionals in Ukraine.

**Conclusion:**

Transition of the course to local ownership was accomplished in partnership with the Ukrainian Family Medicine Training Center in the Bogomolets National Medical University in Kyiv: LMiH is now certified for continuing medical education credit and offered twice a year by this institution. Lessons learned from this experience provide a roadmap for rapidly increasing access to new knowledge and skills for healthcare workers by adapting existing online resources to local needs; they are used to facilitate rapid expansion of other continuing education offerings in Ukraine: additional online courses from the University of Washington (UW) are planned for adaptation.

## Background

Ukraine, with a twenty six-year history of independence from the former Soviet Union, has been investing in several strategic areas for the country’s future development. Healthcare reform is one and continues to require urgent attention from national decision-makers. With this in mind, several recent publications and conferences investigated the current state of pre-service and in-service medical training in Ukraine [1] and the country’s capacity for producing high quality healthcare managers [2, 3]. The vast majority of these managers have a medical background, starting out as practicing physicians and moving into management positions within their healthcare facilities over time. Few have received formal training in leadership and management of healthcare agencies, but some opportunities for training do exist. The School of Public Health of the National University of Kyiv-Mohyla Academy offers a two-year Master’s degree in Healthcare Management and has certified approximately 200 graduates [4]. Traditional classroom-based courses include content on health economics, healthcare management, logistics and operational management, financial management, marketing, investment management, and health technology assessment.

Distance-based learning approaches hold great potential for pre-service and in-service training in Ukraine given the cost of taking health professionals away from work for education and training [5–7]. Systematic reviews of distance learning have found such programs to be either equivalent to, or, in some cases, more effective than traditional learning [8–11]. In the past, distance learning included radio, television, and print-based materials mailed to students or delivered via compact disks, but today it largely refers to electronic learning, or e-learning, based upon access to the Internet. This publication discusses the process and outcomes of adapting an online course developed by a US academic institution, Leadership and Management in Health (LMiH), to the Ukrainian context, including changes required to accommodate the needs of a Ukrainian learner and ensure fit to the Ukrainian context.

### Ukrainian context of medical practice

Ukraine inherited its healthcare system from the former Soviet Union, which has changed very little since Ukraine’s independence proclamation in 1991 [12]. The Ministry of Health of Ukraine (MOHU) is promoting medical reform to ensure that an updated system meets the current population needs in the country [13]. The main principle of reform is for “money to follow the patient”, where people are free to choose their primary healthcare medical provider from a range of clinics and/or hospitals (private and state owned) and/or private doctors (licensed) with patients’ expenses covered (fully or partially) by government. The “state guaranteed package” will cover 100% of medical expenses within this package – primary and emergency care and medical services and drug costs corresponding to about 80% of the population requests for all citizens of Ukraine. The remainder of medical charges will be covered by other means, including personal funds, optional or mandatory medical insurance, and local budgets in accordance with the pricelist regulated by the Cabinet of Ministers of Ukraine [14–16]. Vulnerable groups – retired, people with special needs, and children – will be 100% sponsored by the state.

As much as 93% of all medical providers belong to the state healthcare system under the MOHU; only 7% of doctors are private practitioners, with over 50% of these being dentists. The majority of doctors in Ukraine are female (~ 60%), with approximately 37% of clinic managers being women. Additionally, a quarter of the Ukrainian medical population is either already retired or about to leave the workforce because of age [17, 18].

Seventeen higher medical education institutions awarding medical degrees and/or providing continuing medical education (CME) services belong to the MOHU [19]. CME requires doctors to maintain and update their clinical and/or managerial skills and knowledge through a three-stage “category” certification system [20]. To work toward a higher “category” or to maintain a current one, in addition to having a certain number of years of clinical experience, doctors need to attend lectures, participate in a medical conference, publish a scientific paper, register a patent, defend an academic degree, complete a blended learning course, etc., to collect points - 60, 70, and 80 for the 2nd, 1st, and highest “category” [21]. Today, in the context of Ukrainian healthcare reform, high quality and speed CME training to develop healthcare facilities’ managerial capacity, especially by means of innovative e-learning technologies, is extremely important as recent CME experience has been previously proven to positively influence the quality of care in Ukraine [22, 23]. Additional face-to-face or distance learning courses for medical workers are needed to ensure optimized patient management, especially in primary healthcare facilities and taking into consideration the reform priorities [24].

### The online course LMiH

The twelve-week online course LMiH was initially developed with support from the US President’s Emergency Plan for AIDS Relief (PEPFAR) and the US Health Resources and Services Administration (HRSA). Designed at the University of Washington (UW) in Seattle, USA, the course combines recorded lectures, required reading, self-assessment exercises, and case studies with a weekly online discussion forum.

The Department of Global Health of the UW has been offering this graduate-level course in English for over a decade. By 2017, nearly 10,000 learners from more than 65 countries had enrolled in this course, with a completion rate averaging 83%. The course consists of recorded lectures, required readings, a weekly online discussion forum, quizzes, self-reflection assignments, and a final verbal presentation. It is targeted to practicing healthcare professionals and public health specialists who already have some experience managing people and who can apply the knowledge acquired to their when facilitating meetings, leading teams, resolving conflicts, using data for decision-making, and representing their organization or cause. It focuses on both personal leadership and practical management skills that help participants succeed in complex health environments [25]. LMiH modules include: 1) The Leading and Managing Framework (from Management Sciences for Health); 2) Building Strong Teams; 3) Supervision and Delegation; 4) Conflict Management; 5) Influence without Authority; 6) Systems Thinking; 7) Finance Systems and Accountability; 8) Using Data for Management Decision-Making; 9) Project Management; and 10) Communicating Effectively. It is delivered using Canvas, a learning management system (LMS) that supports construction of a powerful course website. Each of the ten modules is opened weekly, with an introductory and a summary week bookending the course. Although designed as a fully online course, many participants of this online course from UW join local study groups led by a designated (volunteer) facilitator. This allows participants to discuss course concepts in person and to collectively problem-solve leadership and management challenges that learners are facing on the job. The online discussion forum also facilitates this type of discussion and learning. Final course evaluation by participants is among the course requirements. Submission of the completed Individual Learning Plan and a final verbal presentation are worth 70% of the total grade, with 20% given to weekly quizzes and 10% to online discussion board participation. Upon successful course completion, participants can choose to receive either a print or digital Certificate of Completion from UW.

### Course adaptation for Ukraine: Initiation and scoping

The International Training and Education Center for Health (I-TECH) at UW started its technical assistance project for building clinical and managerial capacity of HIV/AIDS services in Ukraine in 2012. In 2013, I-TECH approached the Ukrainian Family Medicine Training Center (historically known as UCFM, from its previous name Ukrainian Center of Family Medicine) offering partnership and extending its capabilities in medical personnel training design, development, implementation, and evaluation gained through 20+ years of technical assistance around the globe. The UCFM was an ideal candidate for such a partnership as they already were involved into the medical training activities in Ukraine yet could significantly benefit from the methodological point of view when it came to producing new trainings as well as from financial support I-TECH had to offer through the PEPFAR and CDC (Centers for Disease Control and Prevention) funding to fight HIV/AIDS in Ukraine.

The UCFM was founded as a part of Bogomolets National Medical University in Kyiv following the Order №228 of the MOHU from May 24, 2005. Its mission consists in facilitating quality improvement, effectiveness, and availability of medical care for the population of Ukraine through education and training of medical personnel, mainly – primary healthcare practitioners. In 2014, I-TECH Ukraine and UCFM released and piloted its first shared product – five day face-to-face training Preventing HIV Transmission and Drug Addiction for family and general practice physicians. A year later, in 2015, a 400+ page training manual for this course was published and approved by the MOHU. The same year, I-TECH Ukraine and UCFM produced their second collaborative training Use of Narcotic, Psychotropic Substances and their Precursors in Family Medicine and its corresponding training manual, also recommended by the MOHU. Consequently, in 2016, upon having taken the original LMiH course through the technical assistance from I-TECH, the leadership of both UCFM and Bogomolets National Medical University proposed to adapt and institutionalize this course for the Ukrainian audience for CME credits (previously reported successful practice [26]). Resulting from this suggestion, the course was adapted and a cohort of 24 participants successfully completed the first pilot in 2016. The course content was significantly improved based on the first cohort feedback, and, in 2017, another group of 27 participants enrolled into the newly CME-approved course, out of whom 22 successfully received a Certificate of Completion, adding 15 points to their next professional qualification.

## Methods

All learning objectives from the original Leadership and Management in Health course were retained. By the end of the course, learners are expected to be able to:

- Approach management challenges in health settings with core knowledge of and skills in organizational management as a guide;
- Pose meaningful questions about what constitutes effective leadership and management in different cultural and organizational settings;
- Make decisions that balance practical concerns with ethical, legal, and compliance considerations;
- Use core principles and tools from human resource and financial management to address challenges and solve problems;
- Translate insights from self-assessment into personal plans for improving leadership and management skills;
- Identify and consult appropriate sources of data for making sound management decisions;
- Align and motivate individuals, systems, and resources toward a common purpose;
- Design and manage systems that are responsive to national and international requirements and demonstrate accountability to stakeholders;
- Identify monitoring and evaluation methods that answer key questions about programmatic efficiency and effectiveness; and
- Use active participation in class discussion activities, and assignments to form or refine a professional value system.

The adapted version of the course also retained the Canvas LMS and website. The Ukrainian team received technical support from the UW via face-to-face work meetings, multiple Skype calls, and email communication in order to master the curriculum adaptation process in Canvas, including uploading audio slides, subtitling videos, and translating reading assignments and questions for quizzes and discussion forums.

Several aspects of the Ukrainian medical environment were also taken into account during the course adaptation phase: attention to language issues, access to the Internet as well as the Internet speed, computer literacy, acceptance of distance learning, and availability of other online learning opportunities on management and leadership. The aspects of cost-effectiveness and sustainability of the LMiH online course adaptation were also considered.

Participant feedback was requested weekly as well at the very end of the course to track the audience experience and accommodate for additional suggestions. The data was collected via Google Forms (the link to questionnaire was incorporated into the Canvas LMS) and analyzed in MS Excel.

Quantitatively, any value participants extracted from the course was presented as “full value” or “half value”. Full value was assigned when a participant claimed having developed his/her skill because of the course completion with the initial (before the course) self-reported skill development level “1” and final as “3”, i.e. 2 points of change, which is possible maximum value. Half value, however, represented change of less than full value, which is “1”, that could be started with “1” and ended with “2” or started with “2” and ended with “3”. As part of the participants stated some of their skill development levels unchanged after the course, the concept of “benefit” was introduced to denote those participants who did extract either full or half value out of the course.

As some participants responses to closed-ended questions of the surveys suggested “Yes”, “Partially Yes”, or “No” answers, the formula was applied to calculate the “Yes” answer percentage and quantify the results: [(# of “Yes”) – (# of “No”) – (# of “Partially Yes” × 0.5)] / (# of “Yes”) and expressed in percentages.

Basic financial analysis was conducted to compare total costs incurred per similar face-to-face and online course offerings.

## Results

### Lesson 1. Use language conducive to your audience learning

A survey conducted in 2012 demonstrated that only 26% of Ukrainian doctors reported fluency in English, with an additional 17% stating they were able to communicate in English without a dictionary [27]. From these data, it appears that Ukrainian physicians, on average, do not have adequate fluency to benefit the most from an English language course. On the other hand, in 2017 around 68% of all Ukrainians considered Ukrainian to be their native language [28]. As a result, the decision was made to translate all the LMiH course materials to Ukrainian, including subtitles added to the recorded lectures, audio files transcribed, and new readings identified. A separate emphasis was put on the adaptive translation of English idioms and real life examples to ensure clarity of the message to non-English speaking participants.

### Lesson 2. Confirm that your audience can access the internet and is computer literate

Very few studies have investigated access to the Internet among Ukrainian doctors. A study in 2011 found that approximately 82% of physician-respondents had physical access to a computer with an Internet connection; of those, 65% had their own personal computer, 8% could use one at either home or work, and 9% could access the Internet elsewhere [29]. This finding is in agreement with an earlier study in 2009 that found 56–71% (depending on the position of the healthcare worker) had access to the Internet [30]. The same study in 2011 also focused on the computer literacy of Ukrainian doctors. According to the authors, only 5% of respondents, on average, identified themselves as experienced computer users; 49% self-identified as moderate users; 37% as beginners; and 9% as non-users. As noted below in Table 1, computer mastery level was found to be a function of the respondents’ age.

**Table 1:** Computer mastery by respondent’s age

Age group	Experienced user	User	Beginner	Non-user
30 and below	8%	71%	20%	1%
31–40	0%	49%	45%	6%
41–50	0%	34%	50%	16%
50 and above	0%	16%	58%	26%

These data are replicated from a chart and might contain errors ±1% when presented as percentages

Only 8% of those aged 30 and below self-identified as experienced users, and there were no experienced users among those aged 31 and older – the majority were either non-users or beginners, with the percentage of users declining with age from 71 to 16%. In addition, the 2012 reports on estimated average Internet speed among Ukrainian users allow for concluding that streaming video content should not be a problem [31]. With access to computers and computer literacy among Ukrainian doctors expected to have increased significantly since 2011, the decision was made to invest in online learning as a cost effective in-service strategy for delivering CME in a country facing rapid healthcare reform.

### Lesson 3. Make sure your audience is ready to accept the distance learning approach

From the same 2011 study, only 13% of respondents claimed to know anything about distance learning; 52% stated having limited knowledge about it; and 35% had never heard of it before. Approximately 44% of this group was 41–50 years old. When asked about their preferences for receiving CME, 23% in 2011 replied they would opt for a distance learning opportunity; in comparison, 66% voiced preference for a traditional method of teaching involving personal communication with an instructor. While acceptance was low at this time, there was evidence of the potential for change. Fifty-nine per cent of the same interviewees believed they could learn from online approach. More recently, interest in, and access to, distance learning courses has been growing in Ukraine. Several blended learning courses (courses combining a distance learning component with traditional face-to-face interaction) have been designed and implemented for pre- and in-service education programs. For example, Ternopil State Medical University currently offers two distance-based degree programs, a Bachelor’s and a Master’s degree in Nursing [32]. In addition, there are CME blended learning courses available on the Basics of Otorhinolaryngology [33], General Practice – Family Medicine Specialization [34], and Dentistry and Orthodontics [35]. Still, as a 2016 study suggested [36], distance learning remains limited in the country [37] despite its rapidly expanding use in other countries. Based on the cost effectiveness of online learning and its growing influence worldwide, the decision was made to explore further how to adapt existing courses to the Ukrainian context.

### Lesson 4. Check if similar online courses have already been made available

Shupyk National Medical Academy of Postgraduate Education in Ukraine has been offering partial distance learning opportunities on healthcare management, quality assurance, business planning, and legal regulation of medical practice since late 2013 [38]. In addition, Zaporizhzhia State Medical University reported in 2017 that they had implemented a four-month blended learning CME course on Healthcare Management designed specifically for leaders of healthcare facilities [39]. Like LMiH, this course attempts to accommodate the busy schedules of the target audience – only one day a month is required for the face-to-face portion of the course, with the rest delivered online. Unlike LMiH, it requires presence in the classroom at a time of increasing economic and time pressure on healthcare providers, growing traffic congestion in cities, and emerging expectations for CME opportunities related to healthcare reform in Ukraine. These were all considerations in the decision to offer LMiH as the first fully online course on healthcare management and leadership in Ukraine.

### Course adaptation for Ukraine: Implementation and results

#### Lesson 5. Online learning is cost-effective even in resource-limited settings

Despite the fact that initial one-time costs for planning, adaptation, and implementation of the LMiH were significant ($23,000 for the first pilot in 2016 and $17,500 for the second in 2017), they will be amortized over the subsequent offerings as the LMiH content is very stable. Therefore, the cost of following cycles will be minimal in comparison with the face-to-face CME trainings for Ukrainian physicians. Indeed, each face-to-face training expenses range from $6000 to $10,000, depending on the number of students and the location of the trainings, and this amount does not include course development phase. While some costs are recouped through registration fees paid by participants of either type of CME, the e-learning courses are ultimately more cost effective since they can be offered indefinitely (especially such as LMiH, given that its content is very stable) and to large numbers of healthcare professionals at minimal expense. Classroom-based CME is increasingly impractical for other reasons, as well. The unstable economic situation in Ukraine, which is further exacerbated by military actions in the Eastern part of the country, makes it difficult for healthcare workers to break away from their clinical practice in order to travel for training purposes. It is, therefore, expected that distance-based on-line training courses for the Ukrainian CME system will be very much in demand over time.

### Course 2017 participant profile

**Fig. 1:**
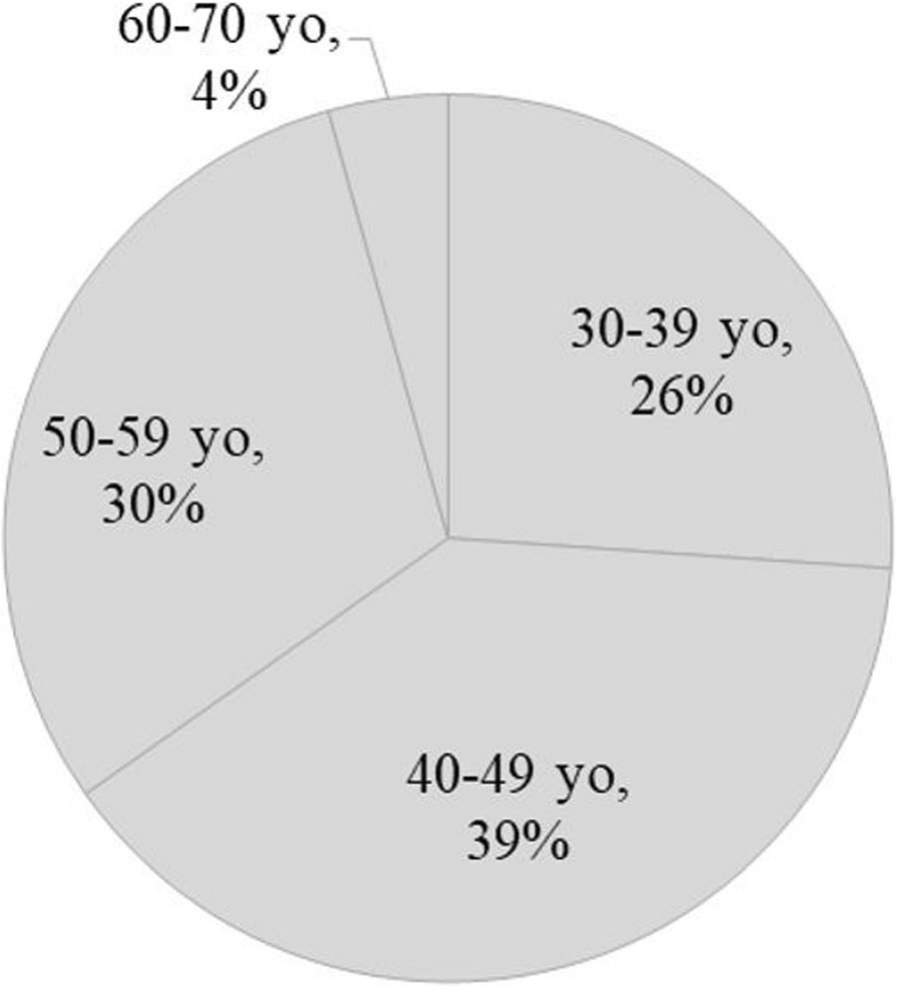
Course participants’ ages. Error of 1% is due to the rounding of numbers. A pie chart representing a distribution of course takers’ ages

The majority (74%) of the 2017 pilot course participants (*N* = 27) were over age 40 (Fig. 1); 16 (59%) were female and 11 (41%) were male.

**Fig. 2:**
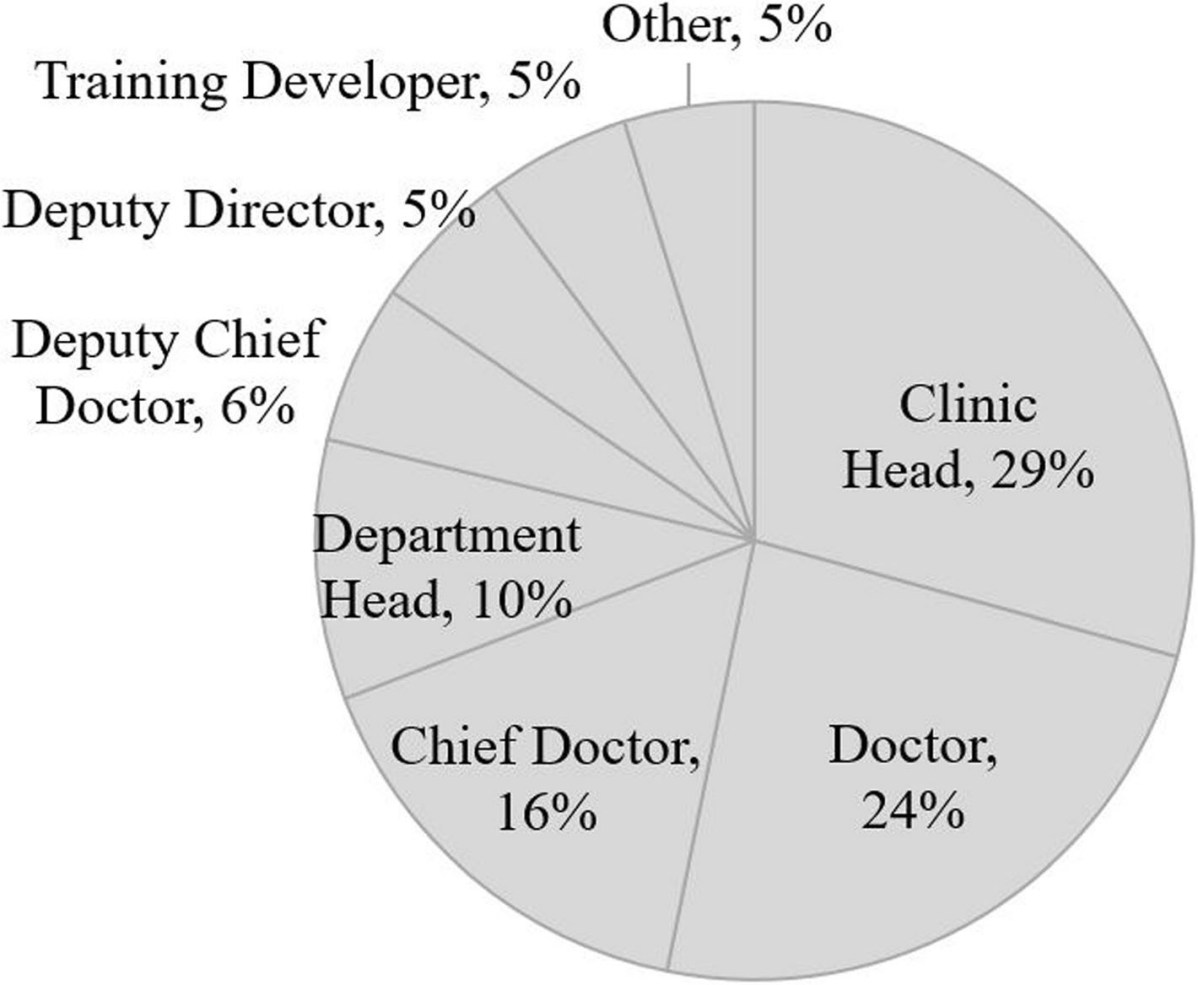
Course participants’ positions. A pie chart representing a distribution of course takers’ positions

The course website site interface was presented in Russian language, since Ukrainian was not available from Canvas. Additional adaptations included adjustments for the Ukrainian context, such as changing cases and assigned readings to healthcare situations more common in Ukraine (i.e., HIV/AIDS or hepatitis C infection instead of tropical diseases). More than 90% of the course participants were physicians and 66% occupied managerial positions, such as Clinic or Department Head, Chief Doctor, or Deputy Director (Fig. 2).

Of all participants in the 2017 pilot course, the majority reported spending, on average, not more than five hours a week on the weekly modules (64%); 32% estimated their time investment as five to ten hours; and less than 4% reported having to study more than 10 h to complete the weekly modules. This correlated well with the estimated 78 h of study stated on the certificate of completion.

### Course 2017 evaluation

On a scale of 1 to 10, participants rated usefulness of all modules as 8.8, with Module #4 (Conflict Management) receiving the highest score (9.4) and Module #8 (Using Data for Management Decision Making) receiving the lowest score of 7.6 (Table 2).

**Table 2:** Survey question: “In general, how useful was this module for you?”. Answers provided on a 1 to 10 scale with 10 being the highest

Module #	1	2	3	4	5	6	7	8	9	10
Average	9.0	9.0	8.8	9.4	9.0	8.5	8.4	7.6	9.0	8.8

Participants may lack experience with making decisions supported by data from human resources, finance, and operations since the Ukrainian healthcare system is still relying heavily on paper documentation rather than upon electronic records that make collated information rapidly available to managers [40, 41].

### Lesson 6. Use local public figures as role models to make the course culturally relevant

Feedback from the initial pilot group in 2016 was favoring following insertion into the course of newly recorded videotaped interviews with recognized Ukrainian medical experts. I-TECH Ukraine ensured balanced representation and voice from the state agencies, health care facilities, and non-profit organizations working in the area of public health. The experts included the Public Health Program Director of the International Renaissance Foundation, both the Development and Innovation Director and Head of the Coordination Council of the All-Ukrainian Network of People Living with HIV, Chief Doctors of the AIDS Centers, representatives of the Department of Public Health and the Public Health Centre of the MOHU, and others. Course participants appreciated these videos more than those of foreign experts (Table 3), with some notable exceptions (Modules #1 [Framework], 7 [Finance] and 9 [Project Management]).

**Table 3:** Survey questions: “Interviews with Ukrainian experts were interesting and informative” and “Interviews with foreign experts were interesting and informative”

# of module	1	2	3	4	5	6	7	8	9	10
% of “Yes”										
Ukrainian	65%	73%	84%	85%	77%	79%	75%	75%	77%	83%
Foreign	83%	64%	68%	83%	71%	45%	80%	58%	81%	82%

Learners rated weekly assignments significantly higher than their experience with the online discussion forums – 87% vs. 66% overall (Table 4). Only Module #8 (Using Data for Decision-Making) scored low for satisfaction with weekly assignments, while the satisfaction scores for several discussion forums were low. For instance, The Leading and Managing Framework; Finance Systems and Accountability; Project Management obtained scores of 62%, 45%, and 50%, respectively. These were the same three modules that scored poorly in terms of Ukrainian vs. foreign expert interviews.

**Table 4:** Survey questions: “Module weekly assignments were interesting to complete” and “Participating in the Discussion Forum was useful and enjoyable”

# of module	1	2	3	4	5	6	7	8	9	10
% of “Yes”										
Weekly assignments	95%	98%	89%	98%	81%	86%	84%	50%	94%	91%
Discussion Forum	62%	75%	71%	73%	80%	67%	45%	75%	50%	65%

This finding can be better understood through participants’ written feedback on module content (e.g., “Finance module was not useful for me since I do not perform financial functions in my job” and “[the] Project Management module was quite complicated; [it] probably should be extended or presented as a separate course”). Project management and financial functions are not presently considered an essential part of healthcare management in Ukraine, though they will be important topics once Ukraine’s healthcare reform is implemented. As for the online discussion forum, it appeared not all respondents felt comfortable discussing work-related issues online since some knew one another through professional networks and could be identified through their names and profile pictures.

Participants did not heavily utilize additional or optional materials made available through the course website. In fact, 35% of the responses claimed no viewing of optional materials at all. Twenty-two per cent reported partial use, and 43% reported full use of additional resource material. Only 7% of participants reported facing technical difficulties when using Canvas, with about half of these complaints related to Module #1 at the beginning of the online course experience. Overall, 80% of participants reported being “very satisfied” and 20% “partially satisfied” with the course. Positive feedback included such statements as: “This course helps to develop practical skills”; “I noticed a change for the better in my professional behavior in a month upon starting this course”; “It was great to have an opportunity to receive this knowledge and build new skills while studying from home and continue working; such information would not be available at any other CME training”; “This course is the best one I have ever attended. The Individual Learning Plan allows for continued development, both personal and professional, even upon official course completion”.

### Value gained

Participants reported between 55 and 95% of benefit gained in a skill development, with the lowest 55% belonging to the category of being better at “supporting my supervisees’ professional development”, “conducting performance reviews” and “listening effectively”. The second lowest score of 65% included being better at “coaching to improve supervisee’s or colleague’s performance”, “knowing my personal communication style”, and “understanding the impact of communication in the workplace”. This might reflect the fact that conducting performance reviews is not common in Ukrainian organizations, and managers normally expect their subordinates to listen effectively instead of being attentive listeners themselves. In other words, course participants might have had difficulty comprehending these concepts and imaging how to effectively apply and benefit from them.

Learners assigned the highest 95% of benefit to skills such as “translating insights from self-assessment into personal plans for improved leadership and management skills”. Each participant was required to complete an Individual Learning Plan and, in doing so, spent a significant amount of time on self-reflection and analysis of their decision-making patterns and leadership styles. Participants reported the highest full value for the following skills: 60% for “identifying characteristics of an effective team” and “distinguishing between task and relationship conflict”, and 55% for “identifying constraints that impact your projects”. All respondents to the final course evaluation confirmed that the course was well organized and that they would recommend it to their colleagues. They reported that written tasks were of an appropriate degree of difficulty, and they were satisfied with the knowledge gained and skills developed. In addition, 95% stated they would use new skills and knowledge in their work. Ninety per cent agreed that weekly quizzes questions were of the right difficulty, and 65% of participants stated that their direct manager was aware of their participation in the course.

## Discussion

The analysis of the two pilots through weekly and final evaluations concluded that the content (building managerial capacity) and approach (online learning) were unique to Ukraine and well accepted by the target audience. All participants of the 2017 pilot reported that they would recommend the course and online format to their colleagues.

### Lesson 7. Have local partners taking ownership over and sustaining the course

The LMiH online course increases the options for professional development available in Ukraine. The course is now fully embraced by the UCFM of the Bogomolets National Medical University in Kyiv, one of the best medical universities in Ukraine [42], is certified for CME credit, and offered twice a year for learners from all over the country. This last point is especially important in the light of the undergoing military conflict with Russia, as even residents of Donbass, where about 30% of medical facilities have been destroyed [43], are able to take part in the course to help maintain their qualifications as needed. Besides, the course is growing in its popularity resulting from the favorable feedback form and good experience of its previous takers: in 2018 the offerings of the LMiH had almost three times more applicants than the course organizers initially planned to accept. The LMiH participants learned to appreciate all the benefits this online course has to offer: 1) its self-paced independent module structure (i.e. can complete separate modules by small chunks and when/where it is most convenient); 2) ability to comfortably combine work and study with no travel required (i.e. no incurred costs of transportation, housing, and out-of-home food); and 3) a unique content normally not widely available in Ukraine for general medical personnel beyond managerial team (i.e. developing competencies immediately needed for work but not present due to the lack of suitable training opportunities and/or specific prerequisites for attendance). Such a success became possible only because of close cooperation of key stakeholders (the MOHU), national level support (the UCFM of the Bogomolets National Medical University), and technical assistance through implementation and beyond (International Training and Education Center for Health in Seattle, Washington, USA and Kyiv, Ukraine).

### Way forward

LMiH is now offered free of charge for representatives of government-owned medical facilities and institutions of higher medical education in Ukraine. Any other organization that wishes to offer the course can admit participants from private medical establishments for a fee of 2000 Ukrainian Hryvnia (UAH), approximately $75 US. Completion of a minimum 75% of all required assignments constitutes the base for learner certification and adds 15 points to professional advancement (“category”).

Adaptation of additional online course offerings from UW is underway applying lessons learned from this effort: language matters; course content is considerably enhanced by engagement of local experts; and computer literacy and access to the Internet are increasing, allowing low cost in-service education to flourish. Further refinements to the LMiH modules on Financial Systems and Accountability and Data for Decision-Making are being made to better reflect conditions in Ukraine, and a separate online course on Project Management in Global Health will be completed by UW and made available to Ukrainian partners for adaptation in 2019.

The limitations of this study is as follow. Ideally, to conclude on cost-effectiveness of the described e-learning course, a similar face-to-face training should be given for the same number of participants to calculate the total cost of attendance and compare with online version financial data. In addition, understanding the reasons behind some participants reporting no development of specific skills after the course completion needs further investigation.

Future research should closely examine the reasons of why course 2017 participants rated Module #8 (Using Data for Management Decision Making) as low as 7.6 out of 10. This will allow for addressing these issues and amending the content to achieve a higher score next time. In addition, investigating experience of those 20% of participants who were “partially satisfied” with the course will provide insights into how to improve it for them. Specifically, the skills with reported low benefits are of interest from this point of view. Finally, it would be helpful to understand why only 65% of participants stated that their direct manager was aware of their participation in the course.

## Conclusions

LMiH was the first fully distance-based e-learning course produced for Ukrainian healthcare workers. The lessons learned during this process of adaptation to a Ukrainian audience are: 1) use language conducive to your audience learning; 2) confirm that your audience can access the Internet and is computer literate; 3) make sure your audience is ready to accept the distance learning approach; 4) check if similar online courses have already been made available; 5) online learning is cost-effective even in resource-limited settings; 6) use local public figures as role models to make the course culturally relevant; 7) have local partners taking ownership over and sustaining the course. The LMiH course is now accredited according to the requirements of the MOHU. Upon completion, all participants receive the appropriate number of credits required for the periodic obligatory recertification of physicians in Ukraine, as well as confirmation of the pedagogical qualifications of medical schools’ and nursing schools’ faculties. Adaptation of additional online courses for Ukraine will benefit from lessons learned through this pilot, further reducing overall costs. This project reflected the values laid out in PEPFAR, specifically, systems strengthening followed by transition to local ownership and technical assistance to assure relevance and long-term sustainability of investments.

## Abbreviations

CDC: Centers for Disease Control and Prevention
CME: Continuing medical education
HRSA: US Health Resources and Services Administration
I-TECH: International Training and Education Center for Health
LMiH: Leadership and Management in Health
LMS: learning management system
MOHU: Ministry of Health of Ukraine
PEPFAR: US President’s Emergency Plan for AIDS Relief
UAH: Ukrainian hryvnia
UCFM: Ukrainian Family Medicine Training Center, previously – Ukrainian Center for Family Medicine
UW: University of Washington
